# Innovative therapeutic strategies to overcome radioresistance in breast cancer

**DOI:** 10.3389/fonc.2024.1379986

**Published:** 2024-05-30

**Authors:** Christoph Reinhold Arnold, Julian Mangesius, Iana Portnaia, Ute Ganswindt, Hendrik Andreas Wolff

**Affiliations:** ^1^Department of Radiology, Nuclear Medicine, and Radiotherapy, Radiology Munich, Munich, Germany; ^2^Department of Radiation-Oncology, Medical University of Innsbruck, Innsbruck, Austria; ^3^Department of Internal Medicine II, Medical University of Innsbruck, Innsbruck, Austria

**Keywords:** radioresistance, breast cancer, lncRNA, miRNA, hyperthermia, nanoparticles, radioimmunotherapy, radiotherapy

## Abstract

Despite a comparatively favorable prognosis relative to other malignancies, breast cancer continues to significantly impact women’s health globally, partly due to its high incidence rate. A critical factor in treatment failure is radiation resistance – the capacity of tumor cells to withstand high doses of ionizing radiation. Advancements in understanding the cellular and molecular mechanisms underlying radioresistance, coupled with enhanced characterization of radioresistant cell clones, are paving the way for the development of novel treatment modalities that hold potential for future clinical application. In the context of combating radioresistance in breast cancer, potential targets of interest include long non-coding RNAs (lncRNAs), micro RNAs (miRNAs), and their associated signaling pathways, along with other signal transduction routes amenable to pharmacological intervention. Furthermore, technical, and methodological innovations, such as the integration of hyperthermia or nanoparticles with radiotherapy, have the potential to enhance treatment responses in patients with radioresistant breast cancer. This review endeavors to provide a comprehensive survey of the current scientific landscape, focusing on novel therapeutic advancements specifically addressing radioresistant breast cancer.

## Introduction

Enhanced understanding of the molecular basis of radioresistance paves the way for novel therapeutic interventions, potentially translatable into clinical applications. This section endeavors to elucidate select promising therapeutic candidates, while recognizing the non-comprehensive nature of this summary. Our focus encompasses three primary research domains: I) modulation of signaling pathways, II) integration of hyperthermia with radiotherapy, and III) incorporation of nanoparticles in oncological treatment strategies. While certain discussed methodologies are currently in various stages of clinical or preclinical implementation, it remains anticipatory to observe additional contenders that may augment the therapeutic outcomes in oncological patients, extending beyond breast cancer.

## Long non-coding RNA and microRNA

Long non-coding RNAs (lncRNAs), structurally similar to mRNA, are composed of more than 200 nucleotides. Unlike mRNA, they cannot produce functional proteins due to the absence of a standard open reading frame (ORF) (1, 2). The 200-nucleotide limit distinguishes lncRNAs from smaller non-coding RNAs such as microRNAs (miRNAs), small interfering RNAs (siRNAs), Piwi-interacting RNAs (piRNAs), small nucleolar RNAs (snoRNAs), and other short RNAs (3). These lncRNAs form complex secondary structures and are tightly regulated. They are crucial in mediating interactions between proteins and nucleic acids, influencing a variety of cellular functions. lncRNAs are primarily involved in gene methylation, initiating transcription, cell cycle regulation, DNA damage repair, and affecting mRNA translation (4). In recent years, advancements in gene sequencing have led to the identification of an increasing number of lncRNAs. Genome-wide association studies on cancerous tissues have revealed several lncRNAs associated with various cancers, including breast cancer, ovarian cancer, and hepatocellular carcinoma (5–8). Moreover, a variety of lncRNAs have been recognized as key players in numerous biological processes, including cell growth, programmed cell death, metastasis, invasion, cell differentiation, chromatin modification, and the movement between the nucleus and cytoplasm.

Recently, a growing body of research has highlighted the critical role played by a particular group of lncRNAs in controlling radioresistance in breast cancer, especially in triple-negative breast cancer (TNBC) (9–11). The control of tumor radioresistance by lncRNAs could include various processes, like the repair of DNA double-strand breaks, apoptosis and autophagy resulting from ionizing radiation (IR), alteration of the cell cycle, and the involvement of the Wnt/β-catenin, PI3K/Akt/mTOR, NOTCH signaling pathways, which are known for their role in cell proliferation and survival (12). Targeting specific lncRNAs that contribute to radioresistance may enhance the efficacy of radiotherapy in BRCA treatment. However, translating these findings into clinical practice requires further investigation, given the complexity of lncRNA interactions and functions and the fact that lncRNAs have only recently become a focus in studies on breast cancer radioresistance. So far, there is an expanding range of preclinical research examining the effects at a cellular level through either the overexpression or suppression of specific lncRNAs.

A recent study discovered lncRNA GAS5 is downregulated in breast cancer and linked to resistance to trastuzumab. A decrease in GAS5 expression was noted in cells exposed to radiation. Overexpression of GAS5 led to a reduction in cell survival, increased apoptosis post-irradiation, and increased G2/M phase arrest along with persistent DNA damage. These findings suggest that GAS5 plays a role in enhancing the sensitivity of breast cancer cells to radiation. Intriguingly, the study also found that miR-21, a miRNA interacting with GAS5, counteracted these radiosensitizing effects by impeding the apoptosis pathway leasing to delayed tumor growth (13).

AFAP1-AS1 is another lncRNA that has been identified as a significant contributor to radioresistance in TNBC, according to a study by Bi et al. This lncRNA plays a crucial role in promoting TNBC’s resistance to radiation by activating the Wnt/β-catenin signaling pathway. Additionally, the study found that suppressing AFAP1-AS1 expression significantly improved the effectiveness of radiotherapy in TNBC tumor models, both xenograft and metastatic (14).

The lncRNA HOX transcript antisense intergenic lncRNA (HOTAIR), hat been shown to negatively regulate radiosensitivity of breast cancer cells and promote their proliferation during radiotherapy (15). Specifically, the upregulation of HOTAIR significantly enhances the growth of MDA-MB-231 and MCF-7 cells following exposure to gamma radiation, and it also increases the survival of MCF-7 cells after irradiation (16). Conversely, knockdown of HOTAIR markedly enhances radiosensitivity and lowers survival rates (17). This phenomenon is mediated through the activation of miR-218, culminating in substantial DNA damage and the initiation of apoptotic pathways. This underscores the potential of HOTAIR as a therapeutic target for augmenting the sensitivity of breast cancer cells to radiation therapy (18). On a mechanistic level, HOTAIR increases the expression of HSPA1A post-transcriptionally. HSPA1A, a key stress-responsive protein in the 70 kDa heat shock protein family, is often overexpressed in various tumors and helps protect cells against environmental stressors (16).

A study by Wang et al. found that suppressing the lncRNA LINC02582 enhances radiosensitivity, whereas its overexpression fosters radioresistance, both *in vitro* and *in vivo* (19). LINC02582, identified as a downstream target of miR-200c (i.e. it can be inhibited by miR-200c), has been shown to exert its role in radioresistance by deubiquitinating and stabilizing checkpoint kinase 1 (CHK1), a key kinase in the DNA damage response. This mechanism suggests that inhibiting CHK1 could be an effective strategy to increase radiosensitivity. Additionally, miR-200c contributes to radiosensitivity by suppressing LINC02582, leading to reduced CHK1 levels and impaired DNA repair.

Lei et al. discovered that the lncRNA DUXAP8 is notably overexpressed in radioresistant breast cancer tissues, correlating with a worse prognosis (20). The study demonstrated that DUXAP8 overexpression contributes to increased radioresistance by activating the PI3K/AKT/mTOR pathway. This pathway is pivotal in cellular signaling, influencing various key biological processes, and playing a significant role in cancer and neurodegenerative diseases (21–23). On the other hand, reducing DUXAP8 expression was found to enhance radiosensitivity, suggesting that targeting DUXAP8 could be a viable strategy for addressing radioresistance in breast cancer.

Like lncRNA, the study of miRNA in the context of breast cancer radioresistance has garnered more focus recently. A recent review identified a variety of miRNAs as key players in regulating the sensitivity and resistance of breast cancer cells to radiotherapy. Notably, among 36 identified miRNAs impacting radio-responses, 22 were found to enhance radiosensitivity, 12 contributed to radioresistance, and 2 demonstrated both effects (24).

An example for miRNA with a dual role is miR-122, which was observed to be upregulated in breast cancer cells with acquired radioresistant (25). In the original, non-resistant cells, miR-122 functions as a tumor suppressor, reducing survival rates and increasing sensitivity to radiation. Interestingly, in cells that have become radioresistant, miR-122 contributes to greater radioresistance by enhancing cell survival. This indicates that miR-122 impacts radiotherapy response differently, possessing a dual functionality that depends on the specific type of cell involved.

Recently, miR-200a has garnered interest due to its role in regulating the transcription factor nuclear factor (erythroid-derived 2)-like 2 (Nrf2) through Keap1. Nrf2 is a central regulator of antioxidant defense systems. In a recent study, it was demonstrated that using shRNA to reduce Nrf2 expression resulted in heightened radiosensitivity in breast cancer stem cells (BCSC), suggesting that miR-200a is a promising candidate for future studies (26).

Lastly, a study by Tomita et al. could show that miR-7–5p was highly expressed in various radioresistant cells, including breast cancer (27). The alteration of miR-7–5p levels played a crucial role in influencing the radiosensitivity of these cells: its overexpression led to increased radioresistance, whereas suppressing miR-7–5p resulted in a decrease in resistance to radiation.

These preclinical investigations underscore the potential utility of lncRNAs and miRNAs in breast cancer treatment, due to their complex and crucial involvement in the modulation of radioresistance in breast cancer cells. **Table 1** summarizes the studies on lncRNA and miRNA that are presented.

**Table 1:** long non-coding RNA and micro-RNA.

	Chromosome location	Target via pathway	Mechanism and effect	Cell lines	References
lncRNA					
Growth arrest-specific transcript 5 (GAS5)	1q25.1	miR-21 via PI3K/AKT/mTOR, miR-34a via NOTCH1 signaling pathway	GAS5 downregulation leads to resistance to trastuzumab and IR. Overexpression of GAS5 suppresses miR-21 expression, leading to upregulation of PTEN and impaired in the Akt pathway. It induces more apoptosis and DNA double-strand breaks (DSBs), leading to delayed tumor cell growth.	MCF-7, MDA231, and MCF-10A	Ma et al. (13)
Actin filament-associated protein 1 antisense RNA1 (AFAP1-AS1)	4p16.1	miR-497–5p, miR-133a-5p via MEK/ERK pathway	AFAP1-AS1 upregulates SEPT2 expression by sponging miR-497–5p, and modulate cell progression. Silencing AFAP1-AS1 inhibited proliferation and migration of breast cancer cells.	Triple-negative breast cancer (TNBC), MDA‐MB‐231	Bi et al. (14)
HOX transcript antisense RNA (HOTAIR)	12q13.13	miR-218, miR-449b-5p, HSPA1A via the targeting of AKT pathway-dependent HOXD10	Inhibition of HOTAIR induced upregulation enhances survival after IR exposure. By suppressing the expression of HOTAIR through siRNA-mediated gene silencing, breast cancer cells exhibit heightened vulnerability to radiation therapy due to induction of DNA damage, cell cycle arrest, and activation of associated pathways, including miR-218.	MCF-7, T47D, LM-MCF-7,34 BT-474, SKBR-3 and MDA-MB-231, TNBC	(15–18)
LINC02582	18q22.3	miR200c via CHK1	Overexpression enhances IR resistance. LINC02582 interacts with the deubiquitinating enzyme ubiquitin specific peptidase 7 (USP7) leading to a critical effector kinase in DNA damage response, promoting radioresistance.	MCF-7, BT474; MDA-MB-231, BT549, SKBR3, T47D	Wang et al. (19)
DUXAP8	20q11.1	miR-423–5p via stimulation of the PI3K/AKT/mTOR pathway and inhibition of EZH2, including RHOB and E-cadherin	The reduction of DUXAP8 enhanced the apoptotic process and increased cell survival following radiation therapy by inhibiting the PI3K/AKT/mTOR pathway.	MCF-12A, MCF-12 F, MCF-7, T47D, ZR-75–1, HCC-1806, MDA-MB-468, BT-549, and MDA-MB-231	(20–23)
miRNA					
miR-122	18q21.31	IGF1R via PI3K/Akt/mTOR/p70S6K	Upregulation of miR-122 suppresses cell growth and cell‐cycle progression and suppressed tumorigenesis by targeting IGF1R and regulating the PI3K/Akt/mTOR/p70S6K pathway inducing cell apoptosis.	TNBC, MCF‐7, MDA‐MB‐231	Perez‐Añorve et al. (25)
miR-200a	1p36.33	Nrf2 via Keap1	Overexpression of miR-200a downregulates Keap1 regulating antioxidant defense systems. Suppressing Nrf2 enhances radiosensitivity in BCSC.	MCF-7, MDA-MB-231	Kamble et al. (26)
miR-7–5p	9q21.32	Proteasome activator subunit 3 (REGγ) via p21 and p53	Through altering the expression of REGγ, the overexpression of miR-7–5p has suppressed proliferation and induced cell death in breast cancer. This member of the REG family depends on p21 and p53 proteolysis for its carcinogenic action.	CRR	Tomita et al. (27)

lncRNA, long non-coding RNA; miR, micro-RNA; IR, irradiation; BCSC, breast cancer stem cells.

Presently, a primary concern in the therapeutic application of long non-coding RNAs (lncRNAs) and microRNAs (miRNAs) is their precise targeting (28). A promising approach to address this challenge involves the knockdown of lncRNA/miRNA utilizing antisense oligonucleotides (ASOs) (29). In a recent study, researchers from Shenzhen demonstrated that targeting LLNLR-299G3.1, a long non-coding RNA (lncRNA) upregulated in esophageal squamous cell carcinoma (ESCC) tissues and cells, which promotes ESCC cell proliferation and invasion, with antisense oligonucleotides significantly inhibited tumor growth and enhanced survival rates *in vivo* (30). In another experimental study, a research team from Gothenburg developed a xenograft model of lung adenocarcinoma. They administered an antisense oligonucleotide (ASO) either peritumorally or systemically to specifically target the long non-coding RNA SCAT7. The intervention led to a significant reduction in tumor growth without inducing observable toxicity (31). However, in a study focused on creating a bioinformatics tool for designing oligonucleotides with tolerable toxicity levels, it was demonstrated that antisense oligonucleotides could induce acute neurotoxic side effects in mice following intracerebroventricular injection (32). In this regard, enhancing the safety of anti-miRNA oligonucleotides can be achieved through chemical modifications that improve the performance and potency of these oligonucleotides (33). Another method for targeting specific microRNAs has been reported by researchers from Jinan, China (34). The team developed a fluorescent nanoprobe designed for the concurrent imaging of miRNA-21 in cancer cells, employing gold nanoparticles as the core and polydopamine as the shell. Confocal microscopy confirmed the efficacy of the nanoprobe for *in situ* monitoring of miRNA-21 and its downstream effects. In a murine model, researchers from Cambridge successfully blocked a critical inducible microRNA expressed in hematopoietic cells, miR-155, utilizing peptide nucleic acids (PNAs). This study highlights an alternative approach for the inhibition of microRNAs (35).

Although clinical data regarding the therapeutic application of lncRNAs and miRNAs in breast cancer remains unavailable, initial early-phase clinical trials targeting other cancer types have recently been documented. For example, H19, a lncRNA characterized by its high diagnostic sensitivity and specificity in breast cancer, has also been identified as overexpressed in pancreatic and bladder cancers. In two clinical trials, patients with unresectable pancreatic cancer, recurrent ovarian/peritoneal cancer, or superficial and intermediate-stage non-muscle invasive bladder cancer were administered BC-819, a plasmid encoding the H19 promoter alongside the diphtheria toxin gene sequence (36–39). The activation of the H19 promoter in tumor cells facilitates the specific expression of the diphtheria toxin within tumor tissues. Consequently, BC-819 has demonstrated efficacy in ablating tumors, reducing tumor growth, extending the duration before recurrence, and exhibiting minimal local toxicity. Nonetheless, the trials were limited by small sample sizes, but clinical phase III trials are planned.

The therapeutic application of lncRNAs or miRNAs presents numerous benefits. First, their tissue-specific and cancer-specific expression profiles make lncRNAs and miRNAs valuable not only for oncological interventions but also for the management of non-oncological diseases. Additionally, these molecules may serve as biomarkers for diagnostic assessments or for monitoring disease progression or therapy efficacy (40–42). lncRNAs and miRNAs are capable of selectively targeting specific genes or pathways, which could result in therapeutic strategies that yield fewer adverse effects compared to conventional chemotherapy (43). Third, it is plausible that lncRNAs and miRNAs could be integrated with other antineoplastic modalities, including radiotherapy, traditional chemotherapy, or immunotherapy. Contemporary therapeutic strategies frequently encompass multiple modalities, each with distinct mechanisms of action, to enhance treatment efficacy (44, 45).

However, alongside the benefits, there are inherent limitations, particularly in the development of pharmaceuticals belonging to an entirely new class. First, although the targeted manipulation of specific lncRNAs is technically achievable, our understanding remains constrained regarding their intricate interactions within the cellular signaling network (46). Additionally, lncRNAs are predominantly nuclear and possess complex secondary structures. Consequently, therapeutics designed to inhibit lncRNAs must penetrate both cellular and nuclear membranes and exhibit sufficient affinity to bind effectively to their target RNAs, presenting significant challenges in drug delivery and binding specificity (47, 48). Finally, it is imperative to consider that, irrespective of preclinical outcomes, clinical trials inherently carry the risk of unexpected events. A notable instance is the CD28 superagonist antibody TGN1412, which, despite auspicious results in cell culture and animal studies, precipitated life-threatening conditions in human subjects (49). Similarly, in 2016, a Phase I clinical trial in France evaluating a novel fatty acid amide hydrolase (FAAH) inhibitor, designated BIA 10–2474, resulted in the death of one participant and severe neurological side effects in others (50). Finally, in the 1990s, a clinical trial garnered significant attention when fialuridine, developed for hepatitis B treatment, induced unforeseen severe liver toxicity. This adverse outcome led to multiple fatalities and necessitated liver transplants for other participants (51). Importantly, in all three instances, the toxic effects were not anticipated in preclinical animal studies. However, vigilance is essential not only in the transition from preclinical to clinical phases but also post-approval, as demonstrated by the unforeseen adverse effects of thalidomide (Contergan), rofecoxib (Vioxx), cerivastatin (Lipobay/Baycol), and trovafloxacin (Trovan/Turvel) (52–55). Following each of these incidents, numerous scholarly articles have been published analyzing the derived lessons. However, it is important to acknowledge that absolute certainty in drug development remains an elusive goal (56–61). Nevertheless, these incidents have prompted the implementation of stricter regulatory measures, which enhance patient safety but also escalate the regulatory challenges associated with the development and approval of treatments based on lncRNAs and miRNAs.

## Hormones, growth factors, and other signaling molecules

Beyond lncRNA and miRNA, a myriad of signaling molecules exert influence on cellular responses to radiation, thereby positioning themselves as potential agents for therapeutic intervention. Subsequent sections will introduce and elaborate on selected examples of these signaling entities.

A study from 2019, initially aimed at creating and analyzing radioresistant breast cancer cell lines, discovered that treating MCF-7 cells with tamoxifen followed by radiation after 24 hours further decreased cell proliferation (62). This suggests that using tamoxifen both before and during radiotherapy in patients with breast cancer could be beneficial.

A different study examined the response to radiotherapy in over 20 breast cancer cell lines, combining this with high-throughput drug-screening data (63). The researchers identified the androgen receptor as a key target for enhancing the effectiveness of radiation therapy. Notably, in TNBC, AR expression was linked to a higher chance of local recurrence following radiotherapy (this was not observed in patients who did not receive radiotherapy), indicating that AR expression could be a useful marker for predicting radiation response in TNBC. Furthermore, blocking the AR with enzalutamide significantly increased radiosensitivity in both *in vitro* and in mice experiments.

The kinase known as Maternal Embryonic Leucine Zipper Kinase (MELK) could be an effective focus for targeted therapies. It has been noted that MELK expression is increased in breast cancer cells, particularly in TNBC, when compared to non-TNBC cells (64). This research highlighted that an increase in MELK expression is a key factor predicting greater radioresistance and higher rates of local recurrence, as evidenced by Kaplan-Meier survival and multivariate analyses across various independent datasets. Targeting MELK, both genetically and pharmacologically, has been shown to enhance radiation sensitivity *in vitro* and significantly slow down tumor progression *in vivo*. This impact is at least partly due to the disruption of non-homologous end joining (65). However, the role of MELK is subject to debate, as indicated by research demonstrating its non-essential nature for the growth of basal-like breast cancer cells (66). Therefore, further investigation into MELK is essential.

Another investigation into TNBC radioresistance revealed that the THO complex (THOC), part of the transcription-export ribonucleoprotein complex that influences the expression of numerous genes and oversees embryonic development, particularly cell proliferation and differentiation, is significantly overexpressed in TNBC’s cancer stem cells (67). Exploring therapies that target THOC may offer novel strategies to tackle cancer stem cells in TNBC. Yet, there has been a lack of additional research published on these proteins.

In an effort to identify new biomarkers for radiosensitivity, a Chinese research team examined all 10 members of the asparagine-linked glycosylation (ALG) family in breast cancer patient samples (68). ALG is a crucial and universally conserved post-translational modification of proteins, vital for precise molecular recognition, protein folding and sorting in the endoplasmic reticulum, intercellular communication, and stability. This study discovered that ALG3 was notably overexpressed in radioresistant breast cancer tissues and was associated with unfavorable clinical and pathological features, as well as poorer overall and local recurrence-free survival rates. Consequently, ALG3 could be a promising biomarker for predicting radiosensitivity and might also be targeted as a radiation sensitizer to enhance treatment response in breast cancer patients exhibiting high levels of ALG3.

In a 2013 study conducted by a San Francisco-based team, it was demonstrated using three-dimensional cell culture that β1 integrin is upregulated through NFkB activation following irradiation (69). β1 integrin, a member of the integrin family of transmembrane cell surface receptors, is another intriguing protein with potential as a therapeutic target. Integrins are crucial for facilitating interactions between cells and the extracellular matrix, and along with other integrin receptors, are known to be overexpressed in various cancers, including breast cancer, where they play a role in resistance to anti-cancer therapies. The observed increase in β1-integrin expression, driven by NF-κB, is closely linked to improved clonogenic survival and tumor regrowth. Therefore, targeting the NF-κB/β1-integrin pathway in radiation-resistant tumors could potentially enhance breast cancer treatments.

The involvement of the tumor microenvironment in radiation resistance has been established, with a recent study from Miami highlighting a significant interaction between cancer-associated fibroblasts (CAF) and breast cancer cells (70). This research demonstrated that luminal breast cancer patient tumors, when irradiated, exhibited increased levels of the NOTCH ligand DLL1. The NOTCH pathway, often altered in cancer, plays a critical role in the self-renewal of CSC, invasion, and the infiltration of various stromal cells in breast cancer. Therefore, radiation exposure might enhance the presence of DLL1+ cancer cells, thereby contributing to radioresistance. Furthermore, DLL1+ cancer cells attract CAFs to the tumor microenvironment through IL-6 secretion. Importantly, both genetic and pharmacological suppression of DLL1 increases the radiosensitivity of cancer cells, particularly when IL-6 is also inhibited, suggesting a new therapeutic avenue.

In a recent study, a Shanghai-based research team conducted a bioinformatic analysis to explore the Gene Expression Omnibus (GEO) database and a survival database for breast cancer patients. They aimed to identify genes linked to poor prognosis following radiation therapy (71). The study found that Growth Differentiation Factor 15 (GDF-15), part of the Transforming Growth Factor Beta (TGF-β) superfamily, was elevated in radioresistant breast cancer cells. This observation aligns with previous findings in human fibroblasts, oral, and lung cancer cells (72–74). GDF-15 is understood to facilitate radioresistance by enhancing epithelial-mesenchymal transition (EMT) properties and stem-like characteristics. Therefore, targeting GDF-15 could present a new avenue for therapy, and the current findings certainly justify additional research in this area.

Reactive oxygen species (ROS) are essential mediators of the cellular impact of radiotherapy, with cancer cells exhibiting low ROS levels, such as cancer stem cells, demonstrating diminished radiosensitivity. An Australian study revealed that following radiotherapy, the integrated stress response (ISR), activated by diverse stressors including glucose scarcity, amino acid shortage, endoplasmic reticulum stress, and hypoxia, emerges as the predominant activated pathway in radioresistant TNBC cells (75). The activation of ISR is a pivotal mechanism in cellular defense against oxidative stress, notably by mitigating ROS accumulation (76). The initiation of ISR involves phosphorylation of eukaryotic Initiation Factor 2α (eIF2α), which subsequently stimulates ATF4 activation and triggers transcription of genes involved in glutathione biosynthesis. This process elevates intracellular reduced glutathione (GSH) levels and enhances ROS scavenging, thereby conferring radioresistance to the cells. Further, inhibiting the eIF2α/ATF4 axis has been demonstrated to reestablish radiosensitivity in radioresistant TNBC both *in vitro* and *in vivo*. These findings underscore the potential of targeting eIF2α/ATF4 signaling as a novel therapeutic approach for TNBC treatment.

In a sophisticated investigation, researchers from California developed a Liquid Chromatography-Parallel-Reaction Monitoring (LC-PRM) methodology for the high-throughput profiling of epitranscriptomic reader, writer, and eraser (RWE) proteins (77). These RWE proteins are integral to the recognition, installation, and removal of modified nucleosides in RNA, playing pivotal roles in RNA processing, splicing, and stabilization. The team employed this technique to quantify these proteins in two sets of matched parental/radioresistant breast cancer cells (specifically, MDA-MB-231 and MCF-7 cells and their respective radioresistant clones), aiming to elucidate the contribution of these proteins to radioresistance mechanisms. The analysis revealed an upregulation of TRMT1, a protein implicated in DNA damage repair, in the radioresistant breast cancer cell lines. Notably, unlike other upregulated proteins, TRMT1’s elevation was significantly associated with reduced survival rates in breast cancer patients who underwent radiation therapy, as evidenced in two patient cohorts, the TCGA-BRCA and METABRIC. Through Gene Set Enrichment Analysis (GSEA), the researchers demonstrated that the DNA repair set was markedly enriched in the cohort expressing high levels of TRMT1. Although the underlying mechanisms remain unclear, TRMT1 emerges as a potential target for enhancing the efficacy of radiation therapy.

Several of these approaches are currently employed clinically, though not specifically aimed at enhancing radiosensitivity, examples include tamoxifen and enzalutamide (78, 79). Although these pharmaceuticals are extensively utilized and generally well-tolerated, it is crucial to acknowledge their associated side effects. Enzalutamide may induce fatigue, diarrhea, neutropenia, hypertension, and headaches, while tamoxifen is linked with cardiovascular, hepatic, and metabolic adverse effects, as well as an increased risk of secondary malignancies (80, 81).. For the other, more experimental methodologies, clinical data are lacking, and several of the previously discussed considerations continue to apply. Modulating signaling pathways for therapeutic interventions, akin to lncRNA and miRNA applications, represents a highly specific form of therapy (i.e. targeted therapies, personalized medicine). This contrasts markedly with conventional, non-specific chemotherapies and may result in fewer adverse effects while enhancing patient quality of life (82, 83). On the downside, many of the aforementioned signaling pathways are incompletely understood, increasing the risk of unwanted side effects, and exhibit redundancy in numerous, if not all, cells, thereby increasing their complexity (84, 85).. Additionally, considering the principles of Darwinian evolution, it is plausible that cancer cells may develop resistance to therapeutics targeting specific signaling pathways through genetic mutations or activation of alternative pathways, similarly to how bacteria acquire resistance to antibiotics (86). Finally, targeted therapies are generally expensive. The high costs are due to several factors, including the complexity of developing these drugs, the extensive research and clinical trials needed to ensure safety and efficacy, and often, the requirement for accompanying diagnostic tests. Additionally, targeted therapies are usually under patent protection, which can limit competition and keep prices high (87, 88).

## Radioimmunotherapy

For many years, the primary goal of radiotherapy has been to enhance local tumor control. The phenomenon known as the abscopal effect, first identified in the 1950s, has received sporadic attention over the years. About two decades ago, foundational research by Sandra Demaria and Silvia Formenti established that T cells are crucial in facilitating the abscopal effect (89). Subsequent to this discovery, the volume of literature on the immune-mediated effects of radiation therapy has surged exponentially. In another significant investigation by Demaria and Formenti, it was demonstrated that the abscopal effect can be enhanced by stimulating the immune system during radiotherapy (90). It is now broadly recognized that radiotherapy can elicit systemic immune responses.

In the early 2010s, the systemic treatment of oncological diseases witnessed a significant advancement with the development of immune checkpoint inhibitors (ICIs). This breakthrough culminated in the awarding of the Nobel Prize in Physiology and Medicine to James P. Ellison and Tasuku Honjo for their discovery of this class of therapeutics (91). Currently, multiple ICIs are available for a wide array of cancer types, with some approved for first-line treatment (92). In summary, ICIs amplify specific immune responses by obstructing inhibitory signals during T-cell activation. Considering that radiotherapy provokes immune responses, the integration of these two therapies is highly rational. Indeed, the combination of immunotherapy and radiotherapy marks a substantial progress in cancer treatment, synergistically improving therapeutic outcomes (93).

Nevertheless, numerous questions remain concerning the optimal integration of ICIs with radiotherapy. Research continues to explore the most effective sequencing of these treatments and the ideal radiation doses and fractionation schemes needed to elicit strong immune responses (94). Moreover, a reevaluation of the strategy to irradiate tumor-draining lymph nodes that are not directly involved may be forthcoming. This common practice is being questioned in light of the sensitivity of T cells to radiation and the critical role of lymph nodes in T-cell activation (95).

For breast cancer, the combination of radiotherapy and ICIs is particularly compelling, given the integral role of radiotherapy in managing this disease. Numerous clinical trials, including randomized phase III studies, are currently investigating how different radiotherapy protocols can be combined with various ICIs, either alone or alongside other systemic treatments like conventional chemotherapy. A comprehensive review detailing the integrated use of radiotherapy and ICIs for treating breast cancer, including an analysis of current trials, has recently been published and is cited herein (96).

## Hyperthermia

Hypoxia, a defining feature of solid tumors, arises due to inadequate vascularization resulting from the aberrant and inefficient neo-angiogenic processes within the tumor microenvironment (97). The neovasculature, originating from the host’s vascular supply, is often rudimentary and disorganized, failing to meet the oxygen requirements of the rapidly proliferating tumor mass. This leads to chronic hypoxia in cells located at the periphery of oxygen diffusion limits. Additionally, the erratic and fluctuating blood flow through these tumor vessels contributes to transient, or acute, hypoxia. Both preclinical and clinical studies have consistently demonstrated that hypoxia within tumors significantly influences malignant progression and the efficacy of treatments, particularly radiation therapy (98). Over recent decades, considerable efforts in both preclinical and clinical research have been directed towards specifically targeting tumor hypoxia to enhance patient outcomes (99). Strategies have included augmenting oxygen supply to the tumor, increasing the radiosensitivity of hypoxic cells, selectively eradicating hypoxic cell populations, and adapting radiation treatment. The latter involves either dose painting, which is the escalation of radiation dose to hypoxic zones, or the use of higher linear energy transfer (LET) radiation, which diminishes the reliance on oxygen for radiation effectiveness (oxygen enhancement ratio or OER). Interestingly, hyperthermia has been identified as a potential comprehensive approach in this context, as it can induce several of the aforementioned effects. Thus, it holds promise as an efficacious modality for the amelioration or eradication of tumor hypoxia.

Hyperthermia therapy, characterized by the elevation of neoplastic tissue temperature to 40–45°C, is acknowledged as an adjunctive modality that enhances the therapeutic efficacy of both radiotherapy and chemotherapy (100). From a biophysical perspective, hyperthermia exerts thermobiological effects leading to the reduction of the α/β ratios in neoplasms, indicative of steepened cell survival curve slopes. At a mechanistic level, hyperthermia-induced perturbations in post-irradiation DNA repair processes result in a preferential enhancement of the quadratic component (β) of the cell kill curve (101). Experimental evidence from *in vitro* studies highlights a diminution in the α/β ratio by approximately 76% (declining from 13.8 to 3.3 Gy) at 41°C and by 37% (from 13.8 to 8.7 Gy) at 43°C (102). The observed reduction at 41°C is primarily attributed to an amplification in the β component, whereas at 43°C, increments in both α and β components were noted, with a more pronounced increase in β. A pivotal investigation in 2019 by a Swiss research consortium assessed α/β ratios derived from a meta-analysis of 12 clinical trials (103). These trials encompassed patient cohorts undergoing radiotherapy or combined radiotherapy and hyperthermia for conditions such as recurrent breast cancer (reBC), locally advanced cervical cancer (LACC), and locally advanced head and neck squamous cell carcinoma (LAHNSCC). The study elucidated that the integration of hyperthermia with radiotherapy notably improved complete response rates, exhibiting α/β ratios of 1.74 for LAHNSCC, 2.05 for reBC, and 3.03 Gy for LACC, and significantly augmented the biological effective dose (BED) from 64.7 Gy to 109.5 Gy. The documented decline in α/β ratios with the addition of hyperthermia to radiotherapy furnishes a scientific rationale for the implementation of hypofractionated treatment schedules. Considering that hyperthermia is conventionally administered once or twice weekly in most treatment facilities, primarily due to the time-intensive nature of the procedure, the synchronization of hypofractionated radiotherapy schedules with hyperthermia sessions could optimize the thermoradiosensitization impact. Furthermore, there is an emerging inclination towards the employment of hypofractionated regimens, especially in palliative care contexts (104). This trend aligns with the strategic objective of maximizing therapeutic efficacy while considering the logistical and physiological constraints of treatment administration.

The augmentation of therapeutic outcomes through the incorporation of hyperthermia alongside radiotherapy and/or chemotherapy has been substantiated in numerous prospective studies, including randomized trials, for a diverse array of malignancies. This encompasses, but is not limited to, carcinomas of the breast, cervix, pancreas, head and neck (specifically squamous cell carcinoma), and rectum (105–113) In cases of recurrent breast cancer, the observed complete response rates to the combination of radiotherapy and hyperthermia exhibit significant heterogeneity, with reported values ranging approximately from 30% to over 90%. A critical determinant hypothesized to account for this broad spectrum of response rates is the inconsistency in hyperthermia administration. This variability encompasses aspects such as the specific hyperthermia methodology employed, the temporal sequencing of radiotherapy and hyperthermia, the duration of the hyperthermia application, and the temperature and thermal dose attained during hyperthermia sessions (114). Furthermore, the evolution of hyperthermia techniques over time has led to disparities in the effective field size and penetration depth, contingent upon both the design and the frequency utilized by the respective technique, thereby influencing the resultant temperature and thermal dose during hyperthermia therapy (115). Ultimately, there is a compelling necessity to establish uniform outcome assessment criteria that extend beyond conventional measures like complete response and overall survival. This is especially pertinent in the context of treatment protocols involving hyperthermia and re-irradiation, where parameters such as tumor dimension should be systematically evaluated (116).

The degree of radiosensitization induced by hyperthermia is markedly contingent upon the sequencing and temporal interval between radiotherapy and hyperthermia, with the most pronounced effect observed when hyperthermia and radiotherapy are administered concomitantly (117, 118). This heightened radiosensitization during simultaneous application is noted in both neoplastic and normal tissues. The literature, encompassing both preclinical and clinical studies, documents a thermal dose-response relationship for hyperthermia (114). *In vitro* investigations reveal an escalation in cellular apoptosis when neoplastic cells are subjected to prolonged or intensified thermal exposure. Numerous clinical studies emphasize the significance of the duration, temperature, and thermal dose attained in hyperthermia treatments. The efficacy of hyperthermia intensifies with extended heating durations with significantly improved clinical outcome, complete response rates, local control and overall survival while also increasing thermal toxicity for patients. Interestingly, exposure to heat can induce thermotolerance, leading to a temporary decrement in heat sensitivity (119). This diminution in hyperthermia efficacy is transient, typically lasting several days. Consequently, clinical applications of hyperthermia are generally restricted to durations of 1–1.5 hours, administered once or twice weekly.

The recent research by the Erlangen group on combining radiation therapy and hyperthermia in breast cancer treatment highlights significant immunobiological implications. Their studies reveal that hyperthermia triggers the release of the immune danger signal HSP70 from breast cancer cells, a response consistent across various temperatures and heating methods (120). Additionally, the combination of hyperthermia and radiotherapy alters the expression of immune checkpoint molecules (ICM) like PD-L1, PD-L2, and others on breast cancer cells. Follow-up studies indicate that the sequence of hyperthermia and radiotherapy doesn’t significantly impact the immune phenotype of breast cancer cells (121). However, the combination leads to an increased expression of immune suppressive checkpoint molecules, suggesting the potential benefit of integrating immune checkpoint inhibitors in multimodal tumor treatments involving radiotherapy and hyperthermia. Future preclinical *in vivo* studies are needed to determine the optimal combination of radiotherapy, hyperthermia, and immune checkpoint inhibition. This research underscores the evolving understanding of how hyperthermia can enhance the efficacy of radiation therapy, particularly from an immunological perspective, offering new avenues for effective breast cancer treatments.

It is pertinent to highlight that the initial capital expenditure required for establishing a hyperthermia treatment facility is comparatively modest (122). This economic feasibility renders hyperthermia therapy a viable therapeutic option not only for smaller, non-hospital-based institutions but also for nations categorized within the low to middle-income bracket.

## Nanotechnology in cancer treatment

Nanotechnology represents a burgeoning domain in oncological diagnostics and therapeutics. Recent evidence underscores its potential to augment the efficacy of radiation therapy. Despite the established effectiveness of radiation therapy in breast cancer management, the collateral toxicity to adjacent healthy tissues remains a significant challenge. Various strategies are under investigation to enhance tumor tissue susceptibility to ionizing radiation via nanoparticle-assisted radiation therapy. This approach aims to surmount radioresistance while concurrently reducing the toxicity associated with the treatment. An overview of selected studies is provided in **Table 2**.

**Table 2:** Nanotechnology in cancer treatment.

	Type of nanoparticles	Targeting	Energy and dose	Size	Outcome	Model
High-Z NP						
Hainfeld et al. (123)	Gold NP	Passive, EPR effect	250 kVp x-rays at 5 Gy min^−1^; 30Gy	1.9 ± 0.1 nm	*In vivo*, one-year survival 86% with gold NRT vs. 20% with IR alone vs. 0% with gold NP alone.	Balb/C mice implanted with EMT-6 syngeneic mammary carcinoma cells
Hainfeld et al. (124)	Gold NP	Passive, EPR effect	30 Gy-42 Gy (68 keV); 44 Gy-60.6Gy (157 keV); 2x15Gy (157 keV) with HT (44 °C for 15 min)	1.9 nm	*In vivo*, long-term survivors 67% with gold-NRT vs. 25% with IR alone. 67% with 68 keV vs. 29% with 157 keV. 75% survivors with gold NRT and hyperthermia vs. 40% with gold NRT alone.	Mouse with xenograft head and neck squamous cell carcinoma
Zheng et al. (125)	Cisplatin-DNA complex gold NP	NA	60 keV electrons	5 ± 2 nm	*In vitro*, 3-fold increase in DSP after IR of DNA-cisplatin-gold NP complexes.	pGEM-3Zf (–) plasmid DNA
Cui et al. (126)	Gold NP	Receptor-mediated endocytosis	225 kVp and 13 mA, 3 x 4 Gy	24 nm	*In vitro* DEFs of 1.25 and 1.14 with gold NP or Cisplatin alone vs 1.39 for combined. *In vivo* increased survival stable intratumoral levels of gold up to 120 h.	Triple-negative breast cancer: MDA-MB-231, xenograft tumors in mice
Mehrnia et al. (127)	AS1411 aptamer-conjugated gold NP	Active, AS1411 aptamer	4 MeV electrons 0–6 Gy	10 nm	*In vitro*, enhanced cell death after IR with targeted gold NP vs non targeted or IR alone.	Breast cancer: MCF-7, MDA-MB-231
Pourshohod et al. (128)	Silver NP conjugated HER2 affibodies	Active, HER2 affibodies	6 MV, 10 Gy photons	120–130 nm	*In vitro*, AgNP-HER2 conjugate enhances cell death after IR vs. non targeted NP or IR alone.	HER2+ breast cancer: SK-BR-3, HN-5, SK-OV-3; breast cancer MCF-7
Chattopadhyay et al. (129)	Trastuzumab conjugated gold NP	Active, HER2 targeting AB	100 kV photons, 11 Gy	30 nm	*In vitro* 3.3-fold increased cytotoxicity with Trastuzumab targeted NP vs. 1.7-fold with non-targeted; *In vivo* 46% tumor volume regression vs. 16% increase.	Breast cancer: MDA-MB-361 xenografts tumor bearing mice
NP influencing ROS production						
Wason et al. (130)	Cerium oxide NP	Passive	160 kV photons, 30 Gy in 5 Gy single doses 3 times weekly over 2 weeks	5–8 nm	Increased ROS production and cell death compared to RT alone, radio sensitization selectively in acidic cells.	Pancreatic tumor (L3.6pl), xenograft tumor murine model
Zhang et al. (131)	Bismuth-NP with S-nitrosothiol	Passive, activated by IR	5 Gy	36 nm	*In vitro* and *in vivo* increased DNA damage with combined bismuth-SNO and IR vs. IR alone.	HeLa, HepG2 cells, U14 tumor bearing mice
Chemotherapy combined NP						
Werner et al. (132)	Folate-targeted docetaxel NP	Active, folate targeted	4 Gy *in vitro*, 12 Gy *in vivo*	72 ± 4 nm	*In vivo*, increased tumor growth delay with targeted docetaxel NP, radiosensitization highly dependent on timing.	KB cells, KB cell xenograft tumors in mice
Other targets						
Gaca et al. (133)	Survivin-miRNA-loaded nanoparticles	NA	6 MeV photons, 2 Gy or 8 Gy	220 nm	*In vitro*, reduction of survivin expression by 50%, increased and cellular toxicity combined with IR.	SW480
Han et al. (134)	Antisense EGFR oligonucleotide nanoparticles	Direct tumor injection	0 – 4 Gy	NA	Increased radiosensitivity, G1 phase arrest, increased apoptotic rate, delayed tumor growth.	SCCVII cells, xenograft tumors in mice

ROS, reactive oxygen species; NP, nanoparticles; IR, irradiation.

## High-Z nanoparticles to increase local radiation energy deposition

A foremost strategy in nanoparticle-mediated radiation therapy involves employing nanoparticles with high atomic numbers (high-Z) to intensify local radiation energy deposition within tumors. Among these, gold nanoparticles have been the subject of extensive research due to their molecular stability and biological inertness. Yet, other elements like hafnium (Hf), bismuth (Bi), gadolinium (Gd), and silver (Ag) also present as viable alternatives. Preclinical investigations indicate that gold nanoparticles, accumulating in tumor tissues through either active or passive targeting mechanisms, can potentiate the efficacy of radiation therapy while mitigating additional toxic effects. A notable *in vivo* study by Hainfeld et al., utilizing a murine model, demonstrated that pre-irradiation administration of gold nanoparticles significantly enhanced tumor regression and survival rates without notable toxicity, in comparison to radiation therapy alone (123). In a subsequent investigation, the group demonstrated that gold nanoparticles could augment the synergistic effects of hyperthermia and radiotherapy (124). The enhanced permeability and retention (EPR) effect is instrumental in the passive accumulation of nanoparticles in tumor tissues, a phenomenon characterized by their extravasation into the tumor interstitium via permeable tumor vasculature (135). Furthermore, the tumor-specificity of nanoparticles can be augmented through active targeting strategies, involving conjugation with tumor-specific markers such as epidermal growth factor receptor (EGFR), human epidermal growth factor receptor-2 (HER2), and angiogenesis markers like the vascular endothelial growth factor receptor (VEGFR) (136).

Gold Nanoparticle Radiotherapy (GNRT) exhibits optimal efficacy with low-energy x-rays and gamma rays (average energy below 100 keV) (137). At these energy levels, photon interaction with gold nanoparticles in tumor tissues predominantly occurs via the photoelectric effect, a critical mechanism for dose enhancement in GNRT. Contrastingly, external beam radiation therapy generally utilizes mega-voltage (MV) radiation, favoring its superior tissue penetration over low-energy photons. Cho et al.’s dosimetric analysis indicated that gold nanoparticles significantly enhance the effectiveness of brachytherapy with low-energy radiation sources like 169Yb, 125I, and 103Pd (138). To adapt the radiosensitizing properties of nanoparticles for MV radiation, more clinically prevalent, Liu et al. proposed the use of silver nanoparticles. These nanoparticles interact with higher-energy radiation primarily through pair production, offering potential improvements in radiation therapy efficacy. However, a major concern regarding the application of metal nanoparticles, including silver, in this context is the potential toxicity due to metal accumulation in healthy tissues. Zheng et al. embarked on a novel oncological treatment approach by integrating cisplatin-based chemotherapy with gold nanoparticles in conjunction with radiation therapy to achieve radiosensitization (125). The simultaneous application of these dual radiosensitizers yielded a remarkable 7.5-fold augmentation in the frequency of DNA double-strand breaks relative to the use of a single sensitizer with radiation, suggesting a synergistic impact in the enhanced disruption of cancer cells.

Cui et al. Investigated the application of gold nanoparticles in the context of TNBC employing both *in vitro* and *in vivo* models (126). Their findings revealed that post-infusion, gold nanoparticle accumulation within tumors remained stable for up to 120 hours. This prolonged stability is critical, indicating the sustained capacity of gold nanoparticles to sensitize cancer cells to radiation over an extended duration. Crucially, their study indicated that this method could potentially amplify the efficacy of cisplatin radiochemotherapy. A pivotal aspect of their findings is that this increased therapeutic effectiveness does not correlate with heightened radiotoxicity.

## Nanoparticles to increase reactive oxygen production

Oxygen scarcity in the tumor microenvironment, particularly in larger lesions, significantly contributes to radioresistance. This is because ionizing radiation primarily damages DNA indirectly through the generation of ROS during water radiolysis. Consequently, nanoparticles engineered to elevate ROS generation could be instrumental in countering radiation resistance, especially in hypoxic tumor regions (139). Cerium oxide nanoparticles demonstrate antioxidative effects in normal cells while exhibiting oxidizing properties in tumor cells. Several studies have identified these nanoparticles as both cytotoxic in tumor cells and potent radiosensitizers, concurrently conferring protection to healthy cells against radiation-induced DNA damage (140–142).

Cerium oxide nanoparticles function as free radical scavengers in neutral pH environments, mitigating superoxide radicals by alternating their valence state from Ce3^+^ to Ce4^+^, and reducing hydrogen peroxide levels through oxidative state modification. Notably, their valence state is regenerative, allowing continuous reaction cycles without depleting the cerium oxide. These nanoparticles have also been observed to upregulate the expression of superoxide dismutase 2, thus offering both direct and indirect protection against radiation-induced free oxygen radicals. In the acidic milieu of hypoxic cancer cells, the catalase-like activity of cerium oxide is inhibited. This leads to the conversion of unstable superoxides into hydrogen peroxide, while further decomposition of H_2_O_2_ is impeded, resulting in its accumulation. Such an accumulation of H_2_O_2_ sensitizes the cell to ionizing radiation (143). In a pancreatic tumor-bearing murine model, pre-radiotherapy administration of cerium oxide nanoparticles significantly enhanced tumor response and augmented apoptotic cell counts compared to radiotherapy alone (130) Therefore, these nanoparticles may concurrently function as both a radiation sensitizer and a radioprotector, presenting novel avenues for cancer radiotherapy. Notably, even at elevated concentrations, these nanoparticles exhibited no toxicity in normal cells (144).

Nitric oxide (NO), known for its vasodilatory properties, can reoxygenate hypoxic tumors, thereby increasing the susceptibility of tumor cells to radiotherapy. However, attaining the requisite concentration for effective radiosensitization *in vivo* is challenging due to vascular activity complications and NO’s brief half-life. Addressing this, Zhang et al. developed a multifaceted bismuth-based nanotheranostic agent, functionalized with S-nitrosothiol. Exposure to XR radiation initiates the breakdown of the S-N bond, triggering a substantial NO release. The study demonstrated that upon 5 Gy irradiation of HepG2 cells, the nanoparticle-induced NO release effectively mitigated radiation and drug resistance (131).

## Active targeting nanoparticles for enhanced specificity

Nanoparticles can be engineered to passively accumulate in tumor tissues via the enhanced permeability and retention effect. However, tumor specificity can be substantially augmented by integrating chemical medicine with tumor-targeting modifications (145). Such design enhancements would result in nanoparticles exhibiting increased radiation-induced cytotoxicity in tumor cells while minimizing accumulation in normal tissues, thereby reducing the required dosage and associated toxicity. A molecularly targeted nanoparticle formulation of docetaxel, utilizing folate as a targeting ligand, has shown notable efficacy as a radiosensitizer, surpassing the effectiveness of both docetaxel alone and non-targeted nanoparticle docetaxel. This effect was found to be highly dependent on the timing relative to irradiation (132). Additionally, gold nanoparticles conjugated with AS1411 aptamers, oligonucleotides that specifically bind to nucleolin receptors often overexpressed in cancer cells, have demonstrated success in *in vitro* experiments on breast cancer cell lines. The employment of AS1411 aptamer-conjugated gold nanoparticles in conjunction with 4 MeV electron beams significantly enhanced radiation-induced cell death, compared to treatments with non-targeted nanoparticles or radiation therapy alone (127).

The utilization of nanotechnology for HER2-positive breast cancer cells has been explored through various methodologies. In an *in vitro* study, the conjugation of silver nanoparticles with ZHER2 affibodies markedly amplified the efficacy of irradiation (using 6 MV LINAC at 10 Gy) relative to either irradiation or nanoparticle (NP) treatment alone (128). Additionally, the conjugation of trastuzumab with 30 nm gold nanoparticles has demonstrated a significant reduction in clonogenic survival following 100 kVp X-ray radiation *in vitro*. This approach also notably enhanced tumor regression post-injection into tumor xenografts compared to irradiation alone. The targeted nanoparticles exhibited increased cytotoxicity (3.3-fold vs 1.7-fold) in comparison to non-targeted gold nanoparticles (129).

## Overcoming radiation resistance with nanoparticles

Acquired radiation resistance in tumors involves various biological pathways, offering numerous targets for nanotechnology-based interventions in radioresistant cancers. Survivin, an inhibitor of apoptosis protein, plays roles in cell division, apoptosis inhibition, cellular stress response, and cell migration. Its overexpression in tumors is associated with resistance to both radiation and chemotherapy (146). A human serum albumin-based nanoparticle system for plasmid-mediated RNA interference targeting survivin has been developed, demonstrating a 50% reduction in survivin expression in SW480 colorectal cancer cells *in vitro* and enhancing cellular toxicity when combined with ionizing radiation (133). EGFR presents another viable target for overcoming radiation resistance. Overexpressed in various cancer types, including TNBC, the anti-tumor efficacy of anti-EGFR treatments is significantly enhanced when combined with radiation, creating a potent synergistic effect. This synergy was investigated using PLGA nanoparticles encapsulated with antisense EGFR oligonucleotides in combination with radiotherapy, focusing on their effect on the radiosensitivity of SCCVII squamous cells. The results showed that antisense EGFR nanoparticles markedly increased radiosensitivity by disrupting EGFR-mediated radioresistance mechanisms. This improvement is particularly advantageous as it facilitates effective cell death in cells typically resistant to either EGFR therapy or radiation alone, thus providing a more comprehensive approach to cancer treatment (134).

## Nanoparticles in clinical trials

Hafnium oxide-based NBTXR3 is a radiation-activated nanoparticle, engineered to augment energy deposition from radiation therapy within tumors. Administered via a single intratumoral injection followed by standard External Beam Radiation Therapy (EBRT), preclinical studies have demonstrated that NBTXR3 can be activated by high-energy photons (1 or 6 MeV), leading to increased local energy deposition in regions containing the nanoparticles. *In vivo* studies showed enhanced radiation response in HT1080 tumor xenografts, with an average dose enhancement factor of 1.5. Moreover, in a HCT116 tumor model in mice, significant survival improvement was observed following NBTXR3 injection and subsequent irradiation with iridium-192, compared to irradiation alone (147). A clinical phase 2/3 trial involving 176 patients with soft tissue sarcoma revealed that the combination of NBTXR3 with external beam radiotherapy (50 Gy over 5 weeks) significantly increased the pathological complete response rate compared to radiotherapy alone (16% vs 8%). Notably, this combination did not introduce new radiation-related toxicities. Adverse events were primarily associated with the injection, involving transient immune reactions, none of which were grade 3 or 4 and were manageable (148). Given its physical mechanism of action, this nanotechnology is anticipated to be effective in a broad range of solid cancers (149).

## Concluding remarks

In conclusion, the integration of nanotechnology, lncRNAs, miRNAs, signaling molecules, and hyperthermia presents a comprehensive strategy to combat radioresistance in breast cancer. Nanotechnology offers targeted drug delivery, enhancing the efficacy of radiotherapy, while the modulation of lncRNAs and miRNAs and various signaling molecules provides a molecular approach to disrupt radioresistant pathways. Hyperthermia serves as an adjunct therapy, sensitizing cancer cells to radiation. Collectively, these innovative approaches herald a new era in personalized and effective treatment strategies for overcoming radioresistance in breast cancer.

## Author contributions

CA: Conceptualization, Data curation, Investigation, Methodology, Writing – original draft, Writing – review & editing. JM: Investigation, Writing – original draft, Writing – review & editing. UG: Supervision, Writing – original draft, Writing – review & editing. HW: Supervision, Writing – original draft, Writing – review & editing. IP: Writing – review & editing.
